# A Narrative Review on Perioperative Pain Management Strategies in Enhanced Recovery Pathways—The Past, Present and Future

**DOI:** 10.3390/jcm10122568

**Published:** 2021-06-10

**Authors:** Qiliang Chen, Erdong Chen, Xiang Qian

**Affiliations:** Department of Anesthesiology, Perioperative and Pain Medicine, Stanford University School of Medicine, Stanford, CA 94305, USA; chenqi@stanford.edu (Q.C.); erdong@stanford.edu (E.C.)

**Keywords:** perioperative pain management, enhanced recovery, personalized medicine

## Abstract

Effective pain management is a key component in the continuum of perioperative care to ensure optimal outcomes for surgical patients. The overutilization of opioids in the past few decades for postoperative pain control has been a major contributor to the current opioid epidemic. Multimodal analgesia (MMA) and enhanced recovery after surgery (ERAS) pathways have been repeatedly shown to significantly improve postoperative outcomes such as pain, function and satisfaction. The current review aims to examine the history of perioperative MMA strategies in ERAS and provide an update with recent evidence. Furthermore, this review details recent advancements in personalized pain medicine. We speculate that the next important step for improving perioperative pain management could be through incorporating these personalized metrics, such as clinical pharmacogenomic testing and patient-reported outcome measurements, into ERAS program.

## 1. Introduction

Effective perioperative pain control is an essential component of surgical recovery [1]. Inadequate pain control is linked to a range of negative consequences. In the immediate postoperative period, poor pain control is associated with a higher incidence of postoperative nausea and vomiting, increased cardiac and pulmonary stress, impaired immune function, delayed wound healing, and increased length of hospital stay [2,3]. It is also a strong predictor of poor long-term outcomes, such as increased psychological stress, delayed ambulation and return of function, higher readmission rate, and overall cost of care [2,3,4,5,6]. Inadequate pain control could also contribute to chronic persistent postsurgical pain (CPPSP), a condition that occurs in 10–30% of postsurgical patients and is defined by pain lasting intraoperative and postoperative pain management can help decrease the physiological and psychological stress response, relieve suffering, and facilitate healing and rehabilitation [7].

Opioid analgesics have been widely used for postoperative pain in the United States (US). Even amidst the current opioid epidemic and emphasis placed on opioid stewardship [8,9,10], US physicians prescribe an excessive amount of opioids to postoperative patients regardless of pain severity or the type of procedures performed [11,12,13]. Lack of proper preoperative pain consultation and education has led to fear of inadequate postoperative control, resulting in patient tendency towards requesting more opioids than they need [14]. Adverse effects associated with overutilization of opioids are well documented, including nausea, ileus, over sedation, respiratory depression, immunosuppression, and rapid development of tolerance, just to name a few [15]. Prolonged opioid use also increases risk of dependence, addiction, and opioid-induced hyperalgesia [16,17]. Understanding these potential risks, the use of opioid analgesics has become more judicious in recent years.

It is also increasingly recognized that social and medical background, pain history, education, psychological status, disease process, and the type of surgery performed all play an important role in individual postoperative pain experience and long-term functional outcome [4,6]. However, this variability and complexity can make evaluating and managing postoperative pain difficult. The current narrative review aims to examine the evidence and provide updates on the current postoperative pain management strategies in ERAS pathways and discuss future directions of personalized pain management.

Literature search was conducted using PubMed, MEDLINE, and Google Scholar database for published articles that have examined perioperative pain management within the past 30 years (1991–2021). Terms used were “ERAS” or “perioperative pain” in combination with “MMA”, “anesthesia”, “personalized medicine”, “patient-reported outcomes” or “pharmacogenomics”. Further articles were found through cross-referencing. Primary studies (e.g., retrospective studies, prospective studies, observational studies, randomized controlled trials etc.), basic science research, metanalyses, and systematic reviews were included, while case reports were excluded from the current review.

## 2. Current Pain Management Strategies in Enhanced Recovery Perioperative Pathways

In response to overutilization of opioids and subsequent poor outcomes, standardization of surgical and anesthetic techniques, perioperative care, and pain management was rapidly popularized in the past decade. Enhanced Recovery After Surgery (ERAS) pathways are comprehensive programs that aim to optimize perioperative care and long-term outcomes [18]. Complementing ERAS programs, a perioperative pain management initiative, PROSPECT, was formed in 2007 by a panel of anesthesiologists and surgeons to provide evidence-based procedure-specific recommendations on postoperative pain control [19,20,21]. Together, they provided guidelines that encompass the entirety of perioperative care and have been shown effective in reducing rates of postoperative complications, opioid consumption, length of hospital stay, and cost in a number of surgical domains [22,23,24,25,26,27,28]. Although opioids remain useful analgesics, an opioid-sparing, multimodal analgesia (MMA) strategy now lies at the center of perioperative pain management within the ERAS guidelines [29]. This includes a range of systemic pharmacological agents as well as targeted analgesia techniques.

### 2.1. Systemic Analgesia Agents

#### 2.1.1. Non-Steroidal Anti-Inflammatory Drugs (NSAIDs)

Medications in the NSAID class, which include cyclooxygenase-1 and -2 (COX-1 and -2) inhibitors, mediate antinociceptive effects by reducing peripheral prostaglandin synthesis and dampening inflammation and swelling associated with tissue damage [30]. NSAIDs are routinely used in ERAS programs, as they are considered potent opioid-sparing analgesics without many undesired side effects, such as nausea or sedation [31]. The recent introduction of celecoxib, a selective COX-2 inhibitor, has significantly decreased the risk of postoperative gastrointestinal bleeding and anastomotic leak that are traditionally associated with non-selective NSAIDs [10]. It is now recommended for perioperative use in most non-cardiac procedures, including spine and orthopedic surgeries [1,10]. Despite their overall favorable safety profile, NSAIDs should be used with caution in patients with impaired renal function due to nephrotoxicity [32].

#### 2.1.2. Acetaminophen

Similar to NSAIDs, intravenous acetaminophen is a potent allosteric COX enzyme inhibitor that has demonstrated opioid-sparing effects when used as part of a multimodal pain regimen [33,34]. Acetaminophen is synergetic with NSAIDs. While NASIDs suppresses peripheral inflammation, acetaminophen’s analgesic and antipyretic effects are secondary to its action in the central nervous system [35]. It is thought that acetaminophen decreases central oxidative stress and prostaglandin release, while engaging descending pain inhibitory pathways [35]. Therefore, ERAS programs continue to strongly recommend co-administration of acetaminophen with NSAIDs perioperatively for optimal pain control [18,30].

#### 2.1.3. Gabapentinoids

Gabapentinoids (e.g., gabapentin and pregabalin) are anticonvulsants that have been used to treat neuropathic pain. Despite their documented sedative and central depressive effects, they are currently strongly recommended in ERAS pathways as earlier randomized controlled trials and meta-analyses demonstrated potent perioperative analgesic and opioid-sparing effects in multiple surgical domains including gynecologic, breast, orthopedic, and spine surgery [36,37,38,39,40,41]. However, the optimal dosage, timing and duration of perioperative gabapenintoid therapy has not been clearly demonstrated, and some argue that clinical effects of gabapentinoids may actually secondary to their sedative properties [42,43]. Recent studies on heterogenous patient populations were unable to demonstrate a clinically significant analgesic effect with perioperative gabapentinoid administration, although they were still effective in promoting opioid cessation compared to controls [44,45]. Future studies will be necessary to further delineate the clinical effects of gabapentinoids and optimize their usage. Overall, current evidence and updated guidelines continue to support the perioperative use of gabapentinoids for their opioid-sparing effects, with close monitoring [1,30].

#### 2.1.4. N-Methyl D-Aspartate (NMDA) Receptor Antagonists

NMDA receptor activation has been implicated in development and maintenance of chronic pain after painful stimuli [46]; thus, receptor antagonists could be desirable as an analgesic and opioid-sparing agent in some perioperative settings [30,43,47]. Intravenous administration of ketamine or magnesium, two commonly used NMDA receptor antagonists, has shown benefit in reduction of postoperative pain and opioid consumption [48,49,50]. Postoperative ketamine infusions at 2 to 5 µg/kg/minute was shown to be effective for pain relief, and reduced opioid consumption by 13 mg morphine equivalents by 48 h [50]. Ketamine also suppresses expression of inflammatory cytokines, such as interlukin-6 and tumor necrosis factor α, which could contribute to analgesic effect [51]. Although routine use of NMDA receptor antagonists is not yet included in most ERAS pathways, they are increasingly recognized as a useful perioperative adjuvant for opioid-tolerant patients and may reduce the incidence of CPPSP [52,53].

#### 2.1.5. Lidocaine

The role of intravenous lidocaine infusion in ERAS pathways remains controversial. The analgesic properties of intravenous lidocaine infusion are still not well understood, but it may be involved in suppression of local and systemic inflammation, nociceptive transmission, and central sensitization [54]. Several randomized controlled trials and meta-analyses have demonstrated that intravenous lidocaine decreases postoperative pain scores, reduces incidence of nausea and vomiting, and promotes return of bowel function in abdominal surgeries [55,56,57,58]. Furthermore, two retrospective analyses have shown that it significantly decreases opioid consumption and has comparable analgesic effect to epidural analgesia in patients with traumatic rib fracture [59,60]. This finding suggests intravenous lidocaine can be a safe alternative in those patients when neuraxial analgesia is not feasible. However, limited by the quality of available data, a recent Cochrane Review by Weibel and colleagues was unable to draw a definitive conclusion on its benefit in perioperative pain management [61]. Therefore, intravenous lidocaine infusion is currently not routinely use for perioperative pain control, and future well-designed trials will be required for delineating its clinical effects and perioperative application.

### 2.2. Locoregional Analgesia Techniques

#### 2.2.1. Neuraxial Analgesia

Epidural infusion of local anesthetic with or without opioids is most commonly used in open thoracic and abdominal procedures and has shown better pain control and lower total opioid requirement than systemic intravenous analgesia alone [29,47,62,63]. Neuraxial analgesia is also effective in reducing perioperative complications, such as ileus, nausea and vomiting, respiratory depression, venous thromboembolism, and arrhythmia, compared to systemic intravenous analgesia alone in both cardiac and non-cardiac patients [64,65]. Some common complications include hypotension, urinary retention, and pruritus (if epidural opioid infusion is used) [64]. For laparoscopic colorectal surgery, epidural analgesia might also prolong length of hospital stay by 1 day when compared to one-time intrathecal analgesia, with no difference in postoperative pain score or complication rate between the two [66]. Overall, neuraxial analgesia is considered safe, and is recommended in many open colorectal, thoracic, gynecologic, and urologic procedures for perioperative pain control by the ERAS society [29,43,47,67].

#### 2.2.2. Regional Analgesia

Regional techniques gained popularity in ERAS pathways in recent years. Transversus abdominis plane (TAP) block has been shown to provide superior pain control and lower perioperative opioid consumption compared to standard intravenous analgesia in both open and laparoscopic abdominal surgeries [68,69,70]. More advanced regional techniques are also being explored. A recent meta-analysis by Liu and colleagues suggested that quadratus lumborum block is more effective at reducing opioid consumption than TAP block for abdominal surgery [71]. Erector spinae plane (ESP) block, a relatively new regional technique that delivers local anesthetics to the dorsal rami and their surrounding structures, has consistently been shown to improve pain control and decrease postoperative opioid consumption [72,73]. It has gained popularity in breast, thoracic, and abdominal surgeries, with ongoing studies exploring utility in other procedures as well [72,73]. Lastly, peripheral nerve blocks such as interscalene, femoral, adductor canal, and sciatic nerve blocks, have also shown similar analgesic and opioid-sparing benefits in various orthopedic procedures (e.g., shoulder surgeries, hip arthroplasty, knee arthroplasty) [47], and are thus recommended in many orthopedic enhanced recovery programs [1,47].

## 3. The Future of Personalized Pain Medicine in Enhanced Recovery Pathways

The enhanced recovery programs have excelled in many traditional clinical outcome measurements including mortality, morbidity, postoperative pain scores, and overall opioid consumption [28,74,75,76,77]. However, it is increasingly recognized that providing personalized, patient-centered care is an essential component in improving patient satisfaction and long-term outcomes [78,79,80]. Integrating personalized pain management strategies throughout the perioperative period could be the next important component of ERAS programs. Although some of these tools, such as personal pharmacogenomics and patient-reported outcome measurements, are novel to most practitioners, they have shown significant potentials to impact clinical decision-making and consensus guidelines on implementation are under continual development and currently available for certain medications. With these patient-specific metrics, pain clinicians can effectively identify and address individual pain concerns, formulate optimal treatment plan, and accelerate rehabilitation and recovery.

### 3.1. Utilizing Pharmacogenomics to Guide Pharmacological Pain Management

MMA has become the centerpiece of perioperative pain management in ERAS programs, but it must be used with caution in medically complex patients. In recent years, there has been burgeoning interest in and recognition of pharmacogenomics influencing personalized pain management, as it could represent an opportunity to identify biomarkers that predict individual pain susceptibility, analgesic response, and drug toxicity [81,82]. The obstacles for implementing pharmacogenomics in pain management are often related to the complexity of and lack of familiarity with genetic testing [83]. Additional challenges are posed by inconsistent results of published studies, availability and cost of reliable genetic testing, and the lack of payer reimbursement structures [83]. As genetic testing becomes more widely available and affordable, it becomes imperative for clinicians involved in treating pain to develop the requisite knowledge and skills to incorporate precision genomic medicine in their preoperative consultation and treatment plan to improve safety and outcomes.

#### 3.1.1. Efficacy of Multimodal Analgesia Can Be Influenced by Genetic Variations

##### Opioid Receptor Mu 1 (OPRM1)

Variations in the OPRM1 gene can potentially influence postoperative opioid response. For incidence, carriers of the OPRM A118G allele were shown to have reduced sensitivity to opioids and presence of the allele is associated with higher postoperative opioid requirement compared to controls [84,85]. Identification of polymorphisms in OPRM1 could provide valuable information on individualized opiate analgesic sensitivity, but more data is need prior to establishment of phenotype based dosing strategies.

##### CYP2D6

Most opioids undergo phase I metabolism in the liver through CYP enzyme catalyzed reactions in which some are converted to active forms while others are inactivated. Codeine is a notable CYP2D6 substrate, and individuals who are poor metabolizers will experience minimal analgesia while ultrarapid metabolizers will be at significant risk for respiratory depression and mortality [86]. This has led the Clinical Pharmacogenetic Implementation Consortium (CPIC) to publish clinical guidance for codeine dosing based on CYP2D6 genotype, recommending that both CYP2D6 ultrarapid and poor metabolizers should not be given codeine [87]. CYP2D6 also metabolizes oxycodone, a commonly utilized opioid, into its more potent metabolite, oxymorphone [88,89]. Thus, slow metabolizers will experience poor analgesia while rapid metabolizers could be at risk for significant adverse effects. Clinically, CYP2D6 polymorphism has also been shown to influence oxycodone metabolism and its analgesic effect in both postsurgical adult and pediatric populations [90,91].

##### CYP3A4

The CYP3A4 gene has also been implicated in metabolism of opioids such as alfentanil, fentanyl, and sufentanil [92,93,94,95,96]. However, studies on CYP3A4 polymorphisms and intraoperative and postoperative opiate requirements have produced inconsistent results and meta-analyses have not yet demonstrated a clear relationship [97,98,99]. Further investigation is needed prior to the creation of formalized clinical guidance.

##### CYP2C9

NSAIDs are recommended in all ERAS pathways for their analgesic effects and minimal potential for addiction but are also associated with potentially deleterious effects on the gastrointestinal, cardiovascular, and renal systems. Multiple studies have linked decreased CYP2C9 function with elevated NSAID exposure and risk of toxicity and adverse events [100,101,102]. The CPIC recently published recommendations and guidelines for initiation and titration of NSAIDs based on individual medical history and CYP2C9 phenotype [103]. Specifically, the lowest normal starting dose of NSAIDs is recommended for patients who have one non-functioning CYP2C9 allele. For the poor metabolizers who are homozygous with two non-functioning CYP2C9 allele, initiating NASID therapy at 25–50% of the lowest normal starting dose is recommended to avoid toxicity [103]. NASIDs with long half-life (i.e., piroxicam and tenoxicam) should also be avoided in the poor CYP2C9 metabolizer population [103].

##### CYP2B6

CYP2B6 is a highly polymorphic gene that is involved in ketamine metabolism, and the interindividual variability in drug response is likely related to CYP2B6 polymorphisms. Presence of the CYP2B6*6 variant allele has been shown in vitro to decrease ketamine clearance [104,105]. Patients with this allele will exhibit substantially decreased steady state plasma clearance and metabolic ratios to ketamine and its metabolites, which could contribute to dysphoric adverse effects [106]. Although no genomic screening guideline for ketamine usage has been published, pain clinicians should be aware of the potential interactions between ketamine and other drugs in patients who are slow metabolizers.

##### Voltage-Gated Sodium-Channel Type IX α Subunit (SCN9A)

Local anesthetics, which function as analgesics by interrupting nociceptive transmission via voltage-gated sodium channel blockade, are often used in neuraxial and regional analgesia as part of multimodal pain regimen in ERAS pathways. SCN9A encodes the Na_v1.7_ sodium channel, and polymorphism of this gene has been shown to affect local anesthetic effects [107,108,109]. Interestingly, loss of function mutation is seen in channelopathy associated insensitivity to pain while gain of function mutation in this gene is also seen in erythermalgia and paroxysmal extreme pain disorder, making genotyping a potentially vital part of perioperative planning in these patients [109,110].

### 3.2. Utilizing Patient-Reported Outcome Measurements to Tailor Pain Management Plan

In addition to the surgical stress, postoperative pain can be impacted by numerous behavioral and environmental factors such as age, past pain history, lifestyle choices, psychiatric comorbidities, and social supports, many of which cannot be addressed with pharmacological therapy alone [111,112]. Recognizing the complexity of perioperative pain and the importance of personalized pain medicine, the joint committee of the American Pain Society, the American Society of Regional Anesthesia and Pain medicine, and the American Society of Anesthesiologists published a clinical practice guideline on perioperative pain management [1]. This guideline highlighted a patient-centered model through which pain management teams establish a shared decision-making process with the patient throughout the perioperative journey [1]. In order to formulate a personalized pain plan, pain clinicians must be able to elicit information from patients and their family regarding to their past experience with pain management, their understanding of the illness, and their goals with surgery. This enables clinicians to provide adequate preemptive psychoeducation for the patients, set reasonable expectations, and address concerns and anxiety [113]. There also needs to be a mechanism for patients to provide feedback throughout the perioperative period for clinicians to adjust the pain regimen and optimize rehabilitation.

Besides traditional objective outcomes (e.g., vital signs, visual pain scale, opioid consumption), assessing subjective patient-reported outcomes, such as pain burden, emotional distress, physical function, and social health allows clinicians to better capture patients’ overall pain experience in response to treatment [114,115]. To meet this demand, the National Institute of Health developed a concise, validated patient-reported outcome assessment tool, the Patient-Reported Outcomes Measurement Information System (PROMIS) (www.nihpromis.org, accessed on 9 June 2021). Nurses, surgeons, anesthesiologists and pain clinicians can us this tool to track these patient-reported outcomes over time [114]. Since its introduction, PROMIS has been proven to be effective and reliable in capturing important subjective health-related quality of life outcomes [116,117,118]. Furthermore, it offers the advantage of allowing patients to input data directly on their own, which minimizes the risk for interpretation bias and lessens the burden on clinical workflow [116,117,118].

Data from PROMIS has been used to improve outcomes in pain patients. The Collaborative Health Outcomes Information Registry (CHOIR), developed by researchers at Stanford University, incorporates PROMIS to help pain clinicians to survey and process patient-reported data from adult and pediatric patients [119,120,121,122,123]. By utilizing a questionnaire from this registry, clinicians are able to identify important physical and psychological risk factors that are predictive of poor pain outcomes [122,123,124,125]. Furthermore, it allows the pain clinicians to allocate resources and individualize treatment strategies for these high-risk patients [123,124,125].

The next step to further improve ERAS pathways could be through implementing the individualized biometrics and patient-reported outcome measurement framework in all phases of perioperative patient care. With personalized data, pain clinicians will be better equipped to manage patients’ expectations, provide psychoeducation, and adjust both pharmacological and non-pharmacological therapies according to functional needs. Integration of patient-reported outcome tracking systems, such as PROMIS and CHOIR, is not only useful in guiding therapy, but pain clinicians can also use this chain of data to track patients’ outpatient rehabilitation and establish follow-up visits if necessary.

## 4. Conclusions

Comprehensive and multimodal pain management is an essential component in the continuum of perioperative care to ensure optimal clinical outcomes for surgical patients. Implementation of MMA strategies and enhanced recovery pathways has repeatedly been shown to improve patient postsurgical outcomes. With increasing efforts being put into providing patient-centered care and personalized medicine in perioperative settings, an evidence-based framework to assess patient-specific data is not only useful, but necessary. Incorporation of clinical pharmacogenomics and patient-reported outcome measurements in treatment planning and shared decision-making can potentially further improve effectiveness and reduce harm in perioperative pain management. Some limitations of these individualized measures include the lack of large-scale randomized clinical studies, unfamiliarity of these tests among clinical practitioners, and uncertainty of best use and integration into regular workflows. Therefore, future research focusing on bridging these knowledge gaps will be important of their eventual implementation. With ongoing efforts on perioperative quality improvement, the field of personalized perioperative pain medicine has tremendous potential.

## Data Availability

No new data were created or analyzed in this study. Data sharing is not applicable to this article.
